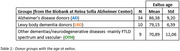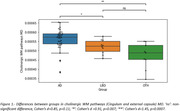# Cholinergic white matter pathways in Alzheimer’s disease, dementia with Lewy bodies, and other neurodegenerative diseases: a post‐mortem MRI study

**DOI:** 10.1002/alz.089110

**Published:** 2025-01-03

**Authors:** Francisco J. López‐González, Milan Nemy, Alberto Rabano, Michel J. Grothe, Pascual Sanchez‐Juan, Daniel Ferreira

**Affiliations:** ^1^ Reina Sofia Alzheimer Center, CIEN Foundation, ISCIII, Madrid Spain; ^2^ Division of Clinical Geriatrics, Center for Alzheimer Research, Department of Neurobiology, Care Sciences and Society, Karolinska Institutet, Stockholm Sweden; ^3^ Reina Sofia Alzheimer Centre, CIEN Foundation, ISCIII, Madrid Spain

## Abstract

**Background:**

In‐vivo magnetic resonance imaging (MRI) has recently shown that patients with clinically diagnosed Alzheimer’s disease (AD) and dementia with Lewy bodies (DLB) exhibit degeneration of the cholinergic nucleus basalis of Meynert and its white matter (WM) projections through the cingulum and external capsule pathways^1^. Here, we propose an imaging‐pathologic validation study aimed at investigating cholinergic WM pathways using post‐mortem MRI of autopsy‐confirmed AD, Lewy body dementia (LBD), and other neurodegenerative diseases (OTH).

**Method:**

We included 53 brain donors (34 AD, 10 LBD, and 9 OTH, mainly including frontotemporal lobe degeneration and vascular disease, Table 1). All donors underwent post‐mortem MRI in situ within 5 hours after death and were later subjected to neuropathological examination performed according to published consensus criteria. Post‐mortem MRI examination included 3D T1 structural imaging and diffusion‐weighted imaging acquired using a 3T scanner. Previously established masks of cholinergic WM pathways (i.e., cingulum and external capsule pathways) were transformed from standard MNI space to each individual’s b0 image in native space using the nonlinear SyN registration algorithm. Native space mean diffusivity (MD) maps were estimated for each donor using the FMRIB Diffusion Toolbox from FSL. Microstructural properties of each participant’s cholinergic WM pathway were then estimated by averaging the MD values within the back‐transformed masks in native space. Differences in age‐adjusted MD values between diagnostic groups were analysed using the Mann‐Whitney U‐test.

**Result:**

AD donors were older than LBD donors (p = 0.007), and both groups were older than the OTH group. After adjusting for age, AD showed significantly higher MD values in cholinergic WM pathways when compared with OTH (Cohen’s d = 1.45, p<0.001) and LBD (Cohen’s d = 0.91, p<0.01) (Figure 1). Elevated MD values in LBD compared to OTH did not reach statistical significance in this small sample (Cohen’s d = 0.85, p = 0.11).

**Conclusion:**

We expanded previous in‐vivo MRI findings to post‐mortem MRI and confirmed the degeneration of cholinergic WM pathways in AD. Additionally, we highlighted the difference between AD and LBD donors, with a more severe cholinergic pathway degeneration in AD donors.

**References**:

1. Schumacher J, et al. Cholinergic WM pathways in dementia with Lewy bodies and AD. Brain. 2022 Jun 3;145(5):1773‐1784.